# Participant Blinding and Gastrointestinal Illness in a Randomized, Controlled Trial of an In-Home Drinking Water Intervention

**DOI:** 10.3201/eid0801.001481

**Published:** 2002-01

**Authors:** John M. Colford, Judy R. Rees, Timothy J. Wade, Asheena Khalakdina, Joan F. Hilton, Isaac J. Ergas, Susan Burns, Anne Benker, Catherine Ma, Cliff Bowen, Daniel C. Mills, Duc J. Vugia, Dennis D. Juranek, Deborah A. Levy

**Affiliations:** * University of California Berkeley, Berkeley, California, USA; † California Emerging Infections Program, Berkeley, California, USA; ‡ University of California San Francisco, School of Medicine, San Francisco, California, USA §California Department of Health Services, Berkeley, California, USA; ¶ Centers for Disease Control and Prevention, Atlanta, Georgia, USA

**Keywords:** bias, epidemiology, double-blind method, filtration, gastrointestinal diseases, public health, randomized controlled trials, standards, sanitation, water microbiology, water supply

## Abstract

We conducted a randomized, triple-blinded home drinking water intervention trial to determine if a large study could be undertaken while successfully blinding participants. Households were randomized 50:50 to use externally identical active or sham treatment devices. We measured the effectiveness of blinding of participants by using a published blinding index in which values >0.5 indicate successful blinding. The principal health outcome measured was “highly credible gastrointestinal illness” (HCGI). Participants (n=236) from 77 households were successfully blinded to their treatment assignment. At the end of the study, the blinding index was 0.64 (95% confidence interval 0.51-0.78). There were 103 episodes of HCGI during 10,790 person-days at risk in the sham group and 82 episodes during 11,380 person-days at risk in the active treatment group. The incidence rate ratio of disease (adjusted for the clustered sampling) was 1.32 (95% CI 0.75, 2.33) and the attributable risk was 0.24 (95% CI -0.33, 0.57). These data confirm that participants can be successfully blinded to treatment group assignment during a randomized trial of an in-home drinking water intervention.

In 1991, Payment and colleagues described a randomized, controlled intervention trial designed to evaluate whether the consumption of tap water treated conventionally to meet regulatory standards affects incidence of gastrointestinal (GI) illness (*1*). In this trial, reverse osmosis filters were installed in 299 households (1,206 persons), and another 307 households (1,202 persons) were followed as controls, with no device installed. After prospective follow-up over a 15-month period, the investigators concluded that 35% of the self-reported GI illness was attributable to tap water consumption. A second trial conducted several years later included treatment groups receiving regular tap water, tap water from a continuously purged tap; bottled treatment plant water; and purified bottled plant water (*2*). This second study attributed 14% to 40% of GI illness to consumption of tap water that met Canadian water treatment standards. Because participants in these studies were not blinded to their treatment group assignments, GI illness may have been overreported by subjects in the tap water groups.

In 1996, the Safe Drinking Water Act of 1974 (*3*) was amended to require the Centers for Disease Control and Prevention (CDC) and the Environmental Protection Agency (EPA) to provide a national estimate of waterborne infectious disease in the United States. In the late 1990s, these agencies funded a large, randomized trial to evaluate the risk for GI illness from the consumption of tap water treated to meet all federal drinking water standards. As a preliminary step in the determination of the national estimate, CDC and EPA funded this pilot study to determine the feasibility of water intervention trials blinding participants to group assignment.

We report the results of the Pilot Water Evaluation Trial (Pilot WET), a randomized, controlled, triple-blinded intervention trial performed in 1999 in households in Contra Costa County in northern California. The primary objective of the trial was to assess whether, for 4 months, participants could be successfully blinded to group assignment, a (sham or active) water treatment device installed underneath the kitchen sink. Secondary objectives included estimating rates of highly credible gastrointestinal illness (HCGI) and other health outcomes and determining the feasibility of performing a similar trial on a larger scale.

## Methods

The study and the informed consent process were reviewed, approved, and monitored by six Institutional Review Boards (Human Subjects Protection Committees) from the investigators’ institutions (University of California, Berkeley; the University of California, San Francisco; the California Department of Health Services; and Public Health Foundation Enterprises, Inc.) and the funding agencies (CDC and EPA).

### Study Area, Water Supply and Water Distribution System

The study area included single-family dwellings served by the Contra Costa Water District. The treatment plant serving the study area used standard conventional treatment with chloramination. A new ozonation plant was completed during the study period, so that after May 1999 the water supply was also ozonated. Source water from the San Joaquin River delta contained agricultural and industrial runoff and pathogens, including *Cryptosporidium*. (More detailed water characterization for the district may be found at http://www.epa.gov/enviro/html/icr/utility/report/CA0710003961023144135.html). Nonetheless, the finished water meets all federal and state drinking water treatment standards and requirements.

### Recruitment, Enrollment, and Compensation of Households

Households were recruited by the Survey Research Center at the University of California, Berkeley, through hand delivery of information packets describing the trial, and by telephone recruitment with a reverse directory in the targeted enrollment areas. To be eligible for the trial, families were required to own their homes, use municipal tap water as the principal drinking water source, and have no household members with a serious immunocompromising condition (such as HIV/AIDS or cancer). Households received $40 on enrollment and an additional $160 in installments on the return of completed health diaries throughout the trial. The first device was installed in March 1999 and the final device in October 1999. Each family was asked to participate for 16 weeks.

One member of each household, designated the “index respondent,” was responsible for communications between the household and the Survey Research Center. The index respondent was the adult member of the household who was in the best position to complete health diaries for other household members who were unable to do so. For 16 weeks, the index respondent mailed completed questionnaires every 2 weeks to the Survey Research Center.

### Randomization and Blinding

Two random sequences were generated to allocate households 50:50 to active or sham filtration devices in blocks of 20. Blocking ensured approximate balance in the number of households per device as participants accrued. One study investigator, who remained unblinded throughout the trial and had no role in data analyses, prepared coded labels from the sequences and sent them to the manufacturer; the manufacturer permanently affixed the labels to the devices. All other study investigators, the plumbing contractor who installed the devices, and the study subjects were blinded as to the household device assignment throughout the trial, including the analysis phase, resulting in a triple-blinded trial.

### Statistical Methods: Blinding Index and Sample Size

The sample size requirement was based on the primary aim of the trial: to determine if subjects could be blinded to water filtration device type. The effectiveness of blinding was quantified by the Blinding Index (BI) of James et al. (*4*), which can be expressed as

BI = *p* x (*r*/2) + *q*,where *p* is the proportion who attempt to guess their device assignment, 0 < *p* < 1; *r* is the ratio, among those who attempt guesses, of the proportions of observed and expected guesses; and *q* = 1 - *p* is the proportion of subjects who do not attempt to guess (i.e., report they “don’t know”). When *r*/2 = 0, BI = *q*, and when *r*/2 = 1, BI = 1; thus 0 < BI < 1. If correct guesses are weighted by 0.0 as recommended (4), then *r*/2 depends only on incorrect guesses. If, in addition, the weights and expected proportions are equal for all incorrect guesses, the term *p* x (*r*/2) equals the proportion of incorrect guesses; otherwise, *p* x (*r*/2) approximates the proportion of incorrect guesses. Thus, BI can be the same or nearly the same as the sum of the proportions of incorrect and “don’t know” responses. The expected values used in the ratio *r* are calculated under the hypothesis that assignments and guesses are independent (i.e., the subjects are blinded); under this hypothesis *r*/2 = 0.5, whereas when the observed proportion of incorrect guesses exceeds the expected proportion *r*/2 > 0.5. Assuming that both incorrect and “don’t know” responses are consistent with blinding, James et al. (*4*) suggest that when the BI is >0.5 the subjects have been blinded successfully on average.

We designed the study to test the null hypothesis with a type I error rate of 0.05, a type II error rate of 0.10, and a variance estimated by BI(1 - BI). In simulations, the distribution of the BI was found to be approximately binomial (data not shown), and this distribution was assumed for variance estimation when the necessary sample size was calculated. Assuming an average household size of 2.4 persons, on the basis of census data, and an intrahousehold correlation of 0.60, based on the work of Donner, Birkett, and Buck (*5*), 80 households, 40 per group, were required.

### Active and Sham Water Treatment Devices

Devices for our trial were purchased from Freshwater Systems, Australia, and installed by Assured Water Products, Inc., a licensed plumbing firm based in Contra Costa County. The devices were designed to be externally identical and to differ only in their ability to remove microorganisms from water.

The active water treatment device contained a 1-micron absolute prefilter cartridge and a UV lamp secured in a quartz sleeve that permitted transmission of UV light. The lamp was designed to emit UV light at 254 nm (optimum for disinfection) with a total minimum dose of 38,000 μ watt-sec/cm^2^ to reduce postfiltration bacteria and viruses by >99% *(6)*. The manufacturer provided written certification that the lamp would emit UV light above this level for 1 year.

The sham device contained an empty filter housing and a UV lamp in a glass sleeve that prevented the transmission of UV light to the water. Inside the empty filter housing, a plastic tube was glued to the inlet to circulate incoming water throughout the empty housing tank to prevent stagnation. Both devices had a tamper-proof seal to prevent opening of the filter casing and an alarm that would sound in the event of failure of the UV lamp or power supply. The devices, installed under the kitchen sink on the cold water line, included a separate drinking water tap at the sink. Both devices provided a water flow through the tap of 5 liters per minute. The cost of the water treatment device, including plumbing expenses, was approximately $988 per household.

### Blinding Outcomes

Every 2 weeks, participants aged >12 years were asked to report on a questionnaire one of five possible responses: "It is definitely the active water treatment device;" "It is probably the active water treatment device;" “It is probably not the active water treatment device;” “It is definitely not the active water treatment device;” or “I'm not sure.” To accommodate the blinding index, these responses were collapsed to three categories: “The active device,” “Not the active device,” or “I don’t know.” We report the BI and 95% confidence interval (CI) based on the week 16 responses, both for index respondents alone and for all household respondents. We adjusted the latter CI for the intrahousehold correlation, ρ=0.60, specified a priori. If the correlation were 0.0, no adjustment would be needed and all participants would be independent observations. If the correlation were 1.0, then each household would be treated as only one observation, since all members of a household would be perfectly correlated in their responses. To supplement these analyses, we also tested, via the 95% CI, the within-assignment null hypotheses that the proportions successfully blinded (i.e., those with "don’t know” or incorrect responses) were <0.5. We did not solicit the blinding status of investigators or contractors. We include the analyses showing only the index respondents to represent a situation in which the correlation coefficient is equal to 0.0.

Finally, to evaluate whether unblinding of participants influenced their reporting of HCGI episodes, we stratified by guess group (active, sham, and don’t know) and estimated, within strata, rates and incidence rate ratios (IRR) of HCGI for the sham and active devices. These analyses were performed by using guesses from the end of study (week 16) questionnaire.

### Health Outcomes

Participants aged >12 years were asked to record each day in diaries whether they had symptoms such as nausea, vomiting, diarrhea, abdominal cramps, cough, and fever; index respondents were asked to record these data for children and other household members who might need assistance. The principal health outcome measured in the trial was similar to the “highly credible gastrointestinal illness” reported by Payment et al. A new episode, defined before the analysis was performed, was defined as any of the following four conditions, preceded by at least 6 HCGI-free days: 1) vomiting; 2) watery diarrhea; 3) soft diarrhea and abdominal cramps occurring together on any day; or 4) nausea and abdominal cramps occurring together on any day.

Episodes during the first 6 days of the study were also included, without the restriction of 6 disease-free days before the study. If HCGI information was missing for a particular day, that day was evaluated as HCGI-free for the purpose of identifying subsequent episodes of HCGI. HCGI data were analyzed by Poisson regression adjusted for the intrahousehold correlation introduced by the clustered sampling design. We examined the duration of HCGI episodes, in days, by device. The attributable risk for HCGI from drinking water was calculated as (IRR – 1) / (IRR), where IRR is the incidence rate ratio of the rate of HCGI in the sham group compared with that in the active group.

### Water Consumption

Water consumption was self-reported by using data collected in questions inserted into the final health questionnaire. Participants were asked to estimate (in numbers of 8-oz. glasses) their consumption of drinking water at home (separately through the study device and through all other sources at home) and outside the home. Participants were provided with water bottles and encouraged to carry water from the home device for use when outside the home. Mean water consumption was compared by study group via the two-sample t-test.

## Results

### Recruitment and Enrollment

Flyers describing the trial were distributed to 29,415 homes. Of 573 households screened after contacting us for more information, 439 (77%) were ineligible for the trial. The most common reasons for ineligibility included using bottled water (21%) or a home water filter device (13%); no children in the household (17%); and preexisting problems with the kitchen plumbing (14%). Of the 134 eligible households, 47 (35%) declined to participate. We were able to install a treatment device in 80 (92%) of the 87 consenting households. Eighty households were needed to meet the sample size requirements discussed below.

Three households were excluded from the trial after the device was installed: one operated a day-care center in the home; at the second, household members objected to the taste of the water after installation; and at the third, household members failed to submit any health diaries. The remaining 77 households (38 active; 39 sham) with 236 participants (118 active; 118 sham) provided partial or complete data on blinding and health outcomes and form the basis for the analyses presented in this report.

### Completeness of Health Data Collection from Participants

For each participant, the maximum number of health diaries that could be collected was eight (biweekly over 16 weeks) with 112 possible days of data (16 weeks times 7 days). Seventy-four (96%) of the 77 households completed all 16 weeks of the trial. In the active group, 879 (85%) biweekly questionnaires were received from a possible 1,032 questionnaires. In the sham group, 861 (89%) of a possible 968 questionnaires were received. In the diaries received, health data were provided for 91% of possible days by participants in the active group and for 86% in the sham group.

### Randomization and Baseline Characteristics (Table 1)

**Table 1:** Baseline characteristics of 236 participants in Pilot Water Evaluation Trial

Characteristic	Sham (n = 118)	Active (n = 118)
Age (Years)	n (%)	n (%)
<11	28 (24)	29 (25)
12-19	10 (8)	13 (11)
20-29	9 (8)	4 (3)
30-39	22 (19)	18 (15)
40-49	21 (18)	24 (20)
50-59	16 (14)	14 (12)
>60	12 (10)	16 (14)
Sex (%)	n (%)	n (%)
Female	57 (48)	56 (48)
Male	61 (52)	62 (52)
Prior medical conditions	n (%)	n (%)
Crohn’s disease	1 (1)	0 (0)
Diverticulitis	1 (1)	3 (3)
Frequent heartburn	5 (4)	8 (7)
Irritable bowel syndrome	7 (6)	2 (2)
Milk intolerance	4 (3)	5 (4)
Stomach ulcer	5 (4)	4 (3)
Ulcerative colitis	0 (0)	1 (1)
Migraine headaches	14 (12)	13 (11)
Self-assessment of current health	n (%)	n (%)
Excellent	42 (36)	41 (35)
Very good	54 (46)	53 (45)
Good	20 (17)	20 (17)
Fair	2 (2)	4 (3)
Poor	0 (0)	0 (0)
Current medical conditions (prior 7 days)	n (%)	n (%)
Abdominal cramps	19 (16)	15 (13)
Diarrhea	14 (12)	13 (11)
Nausea	16 (14)	11 (9)
Vomiting	2 (2)	3 (3)
Fever	6 (5)	5 (4)
Pregnant	1 (1)	1 (1)

The groups were comparable at baseline as measured by the distribution of age, gender, health status, and preexisting gastrointestinal complaints. The average number of participants per household in the sham group was 3.03 and in the active group was 3.11 (p=0.80). The average number of children <12 years of age in each household was 0.73 in the sham group and 0.75 in the active group (p=0.86). Of the index respondents, 67% were female.

### Water Consumption (Exposure) Patterns during the Trial

Participants in the sham group reported drinking an average of 3.1 glasses of unheated water per day from the study device, and those in the active group drank 3.0 glasses per day (p=0.73). There was no difference in the total amount of drinking water consumed by the participants from all sources (mean 6.8 glasses per day in the sham group; 7.4 glasses per day in the active group, p=0.46).

### Effectiveness of Blinding of Participants (Table 2)

**Table 2:** Final (Week 16) Device Blinding Questionnaire, Pilot Water Evaluation Trial

All participants (>12 years of age) who completed final questionnaire			
Guess	Sham device group (%)	Active device group (%)	Total (%)
Sham	12 (17.4)	12 (15.8)	24 (16.6)
Active	30 (43.5)	43 (56.6)	73 (50.3)
Don’t know	27 (39.1)	21 (27.6)	48 (33.1)
Total*	69 (100.0)	76 (100.0)	145 (100.0)

Index respondents only			
Guess	Sham device group (%)	Active device group (%)	Total (%)
Sham	5 (16.1)	5 (15.2)	10 (15.6)
Active	13 (41.9)	19 (57.6)	32 (50.0)
Don’t know	13 (41.9)	9 (27.3)	22 (34.4)
Total	31 (100.0)	33 (100.0)	64 (100.0)

*Does not include 21 participants from the sham device group and 13 participants from the active device group who did not complete the final blinding questionnaire.

+Blinding index for all participants (adjusted for intrahousehold correlation, ρ=0.60)=0.64 (95% confidence interval [CI] 0.51–0.78). Blinding index for index respondents alone = 0.65 (95% CI 0.53–0.76).

The blinding index was 0.64 (95% CI 0.51-0.78) when the week 16 questionnaires of 145 participants >12 years of age were analyzed (Table 2). This finding, adjusted for an intrahousehold correlation (ρ) of 0.60 was highly robust to the choice of correlation coefficients: at ρ=0.40 the CI widens by 0.02, and at ρ=0.80 it narrows by 0.02. The blinding index was 0.65 (95% CI 0.53-0.76) when the 64 index participants were analyzed. Overall, most subjects guessed “active” as their device assignment (50%); 33% guessed “don’t know,” and the rest guessed “sham.”

Within device group, 83% (95% CI 74%-92%) of participants assigned to the sham group appeared to be successfully blinded (i.e., guessed “don’t know” or “active”), compared with 43% (95% CI 32%-54%) of those assigned to the active group. Results among index participants were similar to the overall findings.

### Analysis of Gastrointestinal Illnesses (Tables 3 and 4)

**Table 3:** Episodes^a^ of highly credible gastrointestinal illness (HCGI) and days of illness, Pilot Water Evaluation Trial

	Sham device group	Active device group	Total
Total episodes of HCGI, defined by^b^	103	82	185
Vomiting	18	30	48
Watery diarrhea	73	42	115
Soft diarrhea with abdominal cramps	7	6	13
Nausea with abdominal cramps	16	17	33
Total days of HCGI, defined by^b^	261	190	451
Vomiting	35	78	113
Watery diarrhea	207	99	306
Soft diarrhea with abdominal cramps	8	8	16
Nausea with abdominal cramps	31	30	61
Total days at risk for HCGI episodes	10,790	11,380	22,170
Total days of observation	11,642	12,036	23,678

^a^A new episode was defined as the presence of any of four definitions of HCGI, preceded by 6 HCGI-free days. The difference in total episodes of HCGI was the principal *a priori* health outcome measure for the study.

^b^Because individual participants could report multiple definitions of HCGI on the same day, the total episodes of HCGI (and total days of HCGI) are less than the sums of the individual definitions.

**Table 4:** Rates of highly credible gastrointestinal illness (HCGI) episodes and incidence rate ratios (IRR)^a^ for all respondents and index respondents, by respondent guess about device assignment, week 16,^b^ Pilot Water Evaluation Trial

Guess about device assignment, 16 weeks	All respondents		Index respondents	
	Sham device	Active device	Sham device	Active device
Guess = “Sham”				
Episodes of HCGI	4	4	3	1
Person-time (person-years)	3	3	1	1
No. of respondents	12	12	5	5
Rate (95% CI)	1.2 (0.3–4.8)	1.1 (0.5–2.7)	2.1 (0.7–6.6)	0.7 (0.1–4.8)
IRR for sham vs. active (95% CI)	1.0 (0.2–5.4)		3.16 (0.3–30.4)	
Guess = “Active”				
Episodes of HCGI	30	30	6	15
Person-time (person-years)	8	12	4	5
No. of respondents	30	43	13	19
Rate (95% CI)	3.6 (2.1–6.3)	2.5 (1.3–4.5)	1.6 (0.7–3.6)	2.8 (1.7–4.6)
IRR for sham vs. active (95% CI)	1.5 (0.7–3.3)		0.6 (0.2–1.5)	
Guess = “Don’t know”				
Episodes of HCGI	18	15	10	6
Person-time (person-years)	8	6	4	3
No. of respondents	27	21	13	9
Rate (95% CI)	2.4 (1.2–4.6)	2.5 (1.1–5.5)	2.7 (1.5–5.1)	2.3 (1.0–5.2)
IRR for sham vs.active (95% CI)	1.0 (0.3–2.7)		1.2 (0.4–3.3)	
All guesses				
Episodes of HCGI	52	49	19	22
Person-time (person-years)	19	22	9	9
No. of respondents	69	76	31	33
Rate (95% CI)	2.7 (1.7–4.3)	2.2 (1.5–3.4)	2.2 (1.4–3.4)	2.3 (1.5–3.5)
IRR for sham vs.active (95% CI)	1.2 (0.6–2.2)		0.9 (0.5–1.7)	
No guess given				
Episodes of HCGI	12	5	5	2
Person-time (person-years)	4	2	1	1
No. of respondents	21	13	8	5
Rate (95% CI)	3.3 (1.1–9.7)	2.7 (1.1–6.9)	5.4 (2.6–11.3)	3.0 (0.7–11.8)
IRR for sham vs. active (95% CI)	1.2 (0.3–5.1)		1.8 (0.4–8.8)	

^a^Rates of HCGI and IRR were calculated by Poisson regression and were adjusted for the intrahousehold correlation introduced by the sampling design.

^b^Respondents for the blinding questionnaires were all aged >12 years.

In the sham group there were 103 episodes of HCGI and 10,790 days on which these subjects were at risk for HCGI (3.48 episodes per person-year; adjusted 95% CI 2.26, 5.34). In the active group there were 82 episodes of HCGI during 11,380 days at risk (2.63 episodes per person-year; adjusted 95% CI 1.82, 3.79). The IRR was 1.32 (adjusted 95% CI 0.75, 2.33) when all household respondents were analyzed and 1.09 (95% CI 0.63, 1.90) when data were analyzed only from the index respondent in each household. Data were also analyzed for the component definitions based on the first day of each episode of HCGI (vomiting, watery diarrhea, soft diarrhea with abdominal cramps, and nausea with abdominal cramps) (Table 3). If drinking water were the cause of the reported increase in gastrointestinal disease, the adjusted rate ratio for episodes of HCGI would suggest an attributable risk of 0.24 (95% CI -0.33, 0.57).

HCGI episodes were typically brief; they did not differ in duration between the two groups (p=0.23). The median duration of episodes in the active group was 1 day (range 1 to 40 days; interquartile range 1 to 2 days). The median duration of episodes in the sham group was 2 days (range 1 to 40 days; interquartile range 1 to 3 days)

Among those guessing that they were using a sham device and also among the group of participants guessing “don’t know” the reported rates of HCGI were nearly identical in the two device groups (Table 4). However, among subjects guessing that they were using an active device, the rates of illness were higher among those actually using the sham. A similar pattern (higher rate in the sham group) was seen among subjects who did not complete a final blinding questionnaire.

### Quality Control

Early in the trial we learned that five devices (two active and three sham) had been installed in reverse. The normal flow of water in the device is through the filter first and then through the UV light chamber. In these five devices, the flow passed through the UV chamber first and then through the filter. For all potentially reversed devices (i.e., those installed before the discovery of this reversal), we either directly inspected them or inspected photographs obtained at installation as part of our routine quality control procedures. Although these devices still provided treatment of water, they had not been installed according to protocol and were replaced with identical devices (sham or active) connected correctly. We have retained these households in our analyses.

## Discussion

This pilot study is the first in the United States to evaluate blinding in a randomized, controlled trial of drinking water. Our findings suggest that at least two thirds of participants remained blinded to device assignment throughout the 16-week trial. The actual level of blinding was probably greater, since some subjects may have guessed their device assignment by chance alone.

Our trial was undertaken as the first step in planning a larger trial to evaluate the risk for infection from drinking tap water fully treated to meet conventional regulatory standards in the United States. Without the ability to blind the participants in such intervention trials, the results of any subsequent larger studies intended to evaluate health effects attributable to drinking water would remain controversial. Our data suggest that subjects were effectively blinded throughout the pilot trial. We estimated that a higher proportion of subjects was blinded in the sham group (83%) than in the active group (43%); however, in the active group the 95% CI included 50%, indicating that correct responses may be attributable to chance.

A secondary goal of the trial was to compare gastrointestinal illness rates in the two groups. Although the rate of gastrointestinal illness was higher in the sham group than in the treatment group, this difference was not statistically significant. The relative rates of illness observed overall and in specific subgroups (gender and age) were very similar to those reported in an earlier, larger randomized trial in Canada, which found a statistically significant difference between the active and control groups (1). Preliminary results from a similar trial in Australia, which also was blinded, found no difference in the rates of disease in the active and sham groups (http://www.waterquality.crc.org.au/publictn/wqsmedia.htm).

### Effectiveness of Participant Blinding

Despite the widespread use of participant blinding in intervention trials, little methodologic literature is available with which to measure its effectiveness. In the absence of successful blinding, biases may explain the results of a trial. For example, subjects aware that they are not receiving an intervention (i.e., the sham group) could, intentionally or not, report a higher (or lower) frequency of disease.

Our measurement of blinding is based on work by James (*4*). If 100% of the participants in a study guess their treatment assignment correctly, the BI would be 0.0 (complete unblinding). If 50% of the participants guess correctly, the BI would be 0.5 (random guessing). If 100% of the participants were to guess “don’t know,” the BI would be 1.0 (perfect blinding). Our trial provides evidence that blinding, as measured by the blinding index, can be maintained successfully during an in-home drinking water intervention trial. Subjects in both groups were more likely to guess that they had the active device; we speculate that this may be related to the fact that both active and placebo devices warmed the water during long periods when water was not being drawn from the tap. This warming could have led participants in both groups to believe they were using the active device. Our study lasted only 4 months, and the effectiveness of blinding may decline during a prolonged trial. Drinking water intervention trials conducted for extended periods should also assess blinding throughout the trial. The frequency of such questioning of participants should be designed to avoid raising awareness of treatment group assignment, which might increase unblinding.

### Comparison with Payment’s Prior Intervention Trial

The rates of illness we observed (as measured by HCGI) were higher than those reported in the earlier work of Payment et al. (*1*). Our trial was much shorter than Payment’s (4 months vs. 15 months). Conceivably, subjects in both active and sham groups are more likely to report (or even overreport) illness early in the trial, when enrollment and participation instructions have been recently given and emphasized. Another possible explanation for the difference we observed in the rates of illness between the groups could be that certain persons contributed a disproportionate number of illnesses. However, our data did not support this explanation, since the distribution of number of episodes did not differ between the two groups.

We detected no significant differences in water consumption patterns of the two groups. If any differences in consumption of water outside the home did exist, a conservative bias would have been introduced into our results that would likely have attenuated any difference in observed health effects.

Although our definition of HCGI was patterned after the work of Payment et al. (*1*), there were some differences. In the earlier work, symptoms were reported to the index respondent, who completed all the questionnaires for all subjects. In our study, each participant aged ≥12 years completed his or her own health questionnaires. Payment et al. excluded episodes believed to have other plausible etiologies; we included all episodes. We asked participants to indicate diarrhea on days in which they had two or more loose stools; Payment et al. do not specify the definition of diarrhea. Finally, Payment et al. used the term ”liquid” stool; our term was “watery.”

Payment’s point estimate of the effect (rate ratio = 1.38) is similar to ours (rate ratio = 1.32). Payment reported an attributable fraction of 35% (of HCGI attributable to drinking water consumption); our study’s point estimate of the attributable fraction was 24%.

### Limitations

We selected for the trial only families who owned their homes so that consent would be needed only from the participating family and not also from a landlord. This selection may have led to the recruitment of subjects of higher socioeconomic status than the target population. However, any bias would not affect the internal validity of the study because the subjects were randomized.

Knowledge of experimental group assignment can influence self-reported endpoints in clinical trials, thereby reducing the internal validity of the findings. The experimental group assignment might be revealed to participants through distinguishing features of the intervention (e.g., after installation of the filter, the household water tastes different), through accidental communication of the assignment by study personnel (e.g., the plumber), and, especially in trials with long follow-up, through early or repeated occurrence of an episodic outcome or its symptoms (e.g., HCGI).

Several limitations should be considered in interpreting the health results of this trial. First, it was conducted in a single municipality that received its water from a challenged surface water source and treated water with chloramination. As is typical of randomized, controlled trials, our study relied on volunteers, which hampers external generalizations. As a result of randomization, however, its strength lies in its internal validity (enabling comparison of active and sham groups without fear of selection bias). Data from a series of studies of various designs conducted in various locations are necessary for the development of a national estimate of waterborne disease. This is the approach being used by CDC and EPA. Finally, we provided a treatment device for only one tap in each household. If participants obtained drinking water from other taps (despite our instructions to avoid this as much as possible), our study may underestimate any attributable risk. Use of devices that treated all water entering each household was neither practically nor economically feasible.

Our sample size in this pilot study was determined based on the blinding index. Our study was not designed to be large enough to detect a difference in health (as measured by HCGI) between the sham and active groups of the magnitude previously reported by Payment. If a study were designed with 80% power to detect a true reduction in HCGI to 1.3 episodes/person-year from a level of 2.6 in the sham group, observation of 200 households (of approximatly three persons per household) would be required for one year of observation (based on a two-sided 0.05-level test adjusted for intrahousehold correlation [ρ=0.60]). Additionally, although our study did not collect the data necessary to evaluate the severity of the HCGI episodes, our data indicate that about half the illnesses in both groups were short-lived (only 1 or 2 days long). We suggest that future studies include measurement of episodes associated with lost time at work or school or resulting in calls or visits to physicians, clinics, or emergency rooms. Such measurement will allow better assessment of the public health impact of any differences attributable to drinking water consumption.

One theoretical explanation for the results we observed could be that the sham device somehow degraded the drinking water. In a limited water sampling program (data not shown), we did not find evidence to support this. Additionally, in a large study with the same device in Australia, no difference in health effects was found between the active and sham device groups, suggesting that degradation of the water by the sham device is not a likely explanation for our findings (http://www.waterquality.crc.org.au/publictn/wqsmedia.htm).

Finally, drinking water proceeds in a complicated path from environmental sources, through water treatment and distribution systems, through internal pipes in the home, and eventually to a consumer’s tap. Drinking water intervention trials that use in-home treatment devices cannot isolate the source of any specific site of contamination. Rather, such trials can only help provide evidence to suggest whether further evaluation of the drinking water pathway may be necessary in specific settings.

## Conclusion

Our data suggest that subjects were effectively blinded throughout a 4-month trial of an in-home drinking water intervention. Although the rate of gastrointestinal illness was higher in the sham group than in the treatment group, this difference was not statistically significant, and the trial was not designed to detect a difference of the magnitude observed. The relative rates of illness overall were very similar to those reported in an earlier, larger randomized trial in Canada, which did report statistically significant differences in HCGI between the groups. Our findings suggest that it will be possible to conduct larger blinded, randomized trials to evaluate health effects related to tap water consumption.
